# Body mass index at 11 years and bone mass at age 18: path analysis within the 1993 Pelotas (Brazil) birth cohort study

**DOI:** 10.1186/s12891-015-0529-y

**Published:** 2015-03-29

**Authors:** Ludmila Correa Muniz, Ana Maria Baptista Menezes, Maria Cecília Formoso Assunção, Jeovany Martínez-Mesa, Fernando Cesar Wehrmeister, Laura D Howe, Pedro Curi Hallal, Helen Gonçalves, Fernando C Barros

**Affiliations:** Post-Graduate Program in Epidemiology, Federal University of Pelotas, Rua: Marechal Deodoro 1160 (3° andar). CEP, Pelotas, Rio Grande do Sul 96020-220 Brasil; MRC Integrative Epidemiology Unit at the University of Bristol, School of Social and Community Medicine, University of Bristol, Bristol, UK

**Keywords:** Bone mass, Body composition, DXA, Cohort studies, Adolescence

## Abstract

**Background:**

We investigated whether Body Mass Index (BMI) at 11 years old has a direct effect on bone mass at age 18 operating through alterations to bone growth and development, or whether the association is mediated by concurrent BMI, fat mass (FM), and fat free mass (FFM).

**Methods:**

Path analysis was used to explore the association between BMI at age 11 and whole-body bone mineral content (BMC) and bone mineral density (BMD) assessed by dual-energy x-ray absorptiometry (DXA) at age 18 in a prospective birth cohort study comprising 3,307 adolescents; we also evaluated the degree to which this association was mediated by BMI, FM (kg) and FFM (kg) assessed by plethysmography (BOD POD) at age 18.

**Results:**

We found a positive association between BMI at age 11 and BMC (males [β = 179.7 g, 95% CI 161.4; 198.0]; females [β = 179.9 g, 95% CI 165.3; 194.6]) and BMD (males [β = 0.030 g/cm2, 95% CI 0.024; 0.035]; females [β = 0.029 g/cm2, 95% CI 0.025; 0.033]) at age 18. This association was largely mediated by BMI and FFM at age 18 in both female and male adolescents. FM at age 18 was not an important mediator.

**Conclusions:**

Concurrent BMI and FFM were the main mediators of the association between BMC/BMD in late adolescence and BMI in early adolescence.

**Electronic supplementary material:**

The online version of this article (doi:10.1186/s12891-015-0529-y) contains supplementary material, which is available to authorized users.

## Background

The development of the human skeletal system and bone health are affected by genetic, sociodemographic, hormonal, environmental, and nutritional factors, as well as the interactions among them [1]. Of the measures of nutritional status, body weight has been identified as a major determinant of fracture risk, given its direct association with bone content and bone mineral density (BMD) [2]. Many studies have shown that both high body weight and high body mass index (BMI) are associated with higher bone mass and that weight loss may lead to bone loss [3,4].

It is therefore possible that BMI during childhood and adolescence might influence later BMC/BMD. The existing evidence on this topic is inconsistent. For example, Tandon et al. report positive correlations between BMI from age 4 onwards with both BMC and BMD assessed during adulthood [5], whereas some studies finding excess weight to be associated with lower bone mass [6-8] and others not finding any association between BMI and BMD [9].

The mechanisms underlying a possible association are not clear. One possibility is that BMI during childhood/adolescence, when growth is rapid, leads to immediate changes in the mechanisms underlying bone growth, and these mechanistic changes persist across the life course. Alternatively, concurrent body size may be the most important determinant of BMC/BMD, and any association between BMI in childhood and later BMC/BMD could be due to ‘tracking’ of body size and composition. Prospective studies are needed to disentangle these two potential mechanisms.

Regarding body weight components, El Hage et al. reported that FM was the key determinant of BMD in girls while FFM was the key determinant of BMD in boys pointing to an apparently gender-dependent relationship during adolescence [10].In this study, we assessed the association between BMI at age 11 and BMC and BMD at age 18 in a large prospective cohort from Pelotas, Brazil. We further examined the potential roles of BMI, FM and FFM at age 18 in explaining these associations. Considering that body composition changes between sexes the analysis of the present paper was stratified by sex.

## Methods

All live births in 1993 (N = 5,265) living in the urban area of Pelotas, a southern city in Brazil, were eligible to participate in a cohort study. The cohort sample comprised 5,249 live births (16 refused to participate). We followed subsets of this original cohort at the age of one, three, and six months and one, four, six, and nine years. In 2004–2005, 2008–2009, and 2011–2012, when cohort participants were 11, 15 and 18 years of age, respectively, all participants of the original cohort were invited to follow-up assessments. The analyses of the present study were based on data collected in the follow-ups of the 11 and 18 years old. The full methods of the 1993 Pelotas (Brazil) Birth Cohort Study are published elsewhere [11-13].

The outcomes were whole-body BMC (g) and BMD (g/cm^2^). Both measures were obtained when participants were aged 18 years by dual-energy x-ray absorptiometry (DXA) (Lunar Prodigy Advance – GE®, Germany) [14]. DXA scans were not performed in participants who were pregnant/suspected pregnant, wheelchair users and/or individuals with osteoarticular deformities, those who had implanted metal pins, screws, plates and non-removable metallic objects (body piercings and/or chains), extremely obese individuals, or those with height over 1.92 m.

The main exposure of interest was BMI-for-age (z-score) at age 11 years, defined according to the World Health Organization (WHO) reference charts for children and adolescents 5–19 years [15]. BMI (z-score), FM (kg) and FFM (kg) at age 18 were evaluated as potential mediators of the association between BMI at age 11 and BMC/BMD at age 18. FM and FFM were obtained by air-displacement plethysmography (BOD POD®), since it is considered the gold standard for measuring FM and FFM [16-18]. All variables were analyzed as continuous values.

**Figure 1:**
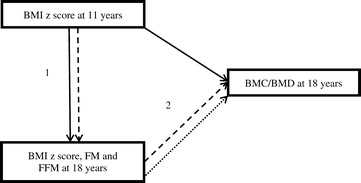
**Analytical model.** Detailed legend: Analytical model to evaluate the association between body mass index (BMI) z score at 11 years and total body bone mineral content (BMC) and body mineral density (BMD) at 18 years old, and the mediation by BMI z score, fat mass (FM) and fat free mass (FFM) at 18 years. Endnotes: 1. Adjusted for skin color, maternal schooling, family income, physical activity and smoking at 11 years; 2. Adjusted for 1 + physical activity, smoking, calcium intake, height and height squared at 18 years. Solid lines: direct effect of BMI z score at 11 years old on BMI z score, fat mass and fat free mass at 18 years and on BMC/BMD at 18 years. Dashed lines: indirect effect of BMI z score at 11 years on BMC/BMD after mediation through BMI z score, fat mass and fat free mass at 18 years (calculated as the product of these two arrows). Dotted lines: direct effect of BMI z score, fat mass and fat free mass at 18 years on BMC/BMD at 18 years old.

Analyses were performed using Stata 12.0 (Stata Corp., College Station, Texas, and EUA) and stratified by sex given evidence of a gender difference in bone mass acquisition [1,19]. We used path analysis (a form of structural equation modelling that facilitates analysis of mediation) to investigate whether the association between BMI at age 11 and BMC/BMD at age 18 is mediated by body composition at age 18, or whether there is a direct effect of BMI at age 11 (i.e. an effect that operates through pathways other than through BMI, FM or FFM at age 18). Figure 1 shows hypothesized relationships between the study variables.

We evaluated the mediating roles of BMI, FM and FFM at 18 years, i.e. contemporaneous to the outcomes separately, and the combined role of these potential mediators. For each model, we calculated the total effect (the overall association between BMI at 11 and BMC/BMD at age 18 after adjustment for confounders), the direct effect (the association between BMI at age 11 and BMC/BMD at age 18 after taking account of both confounders and mediators) and the percentage of the association between BMI at age 11 and BMC/BMD at age 18 that is explained by each mediator (or set of mediators).

At age 11 the following confounders were taken into account: interviewer-reported skin color (white, non-white); family income (in Brazilian Real [RS]); maternal education (0–4, 5–8, 9–11, 12 or more years of schooling); leisure-time and commuting physical activity (minutes/week) and adolescent smoking status (smoked at least one cigarette in the last 30 days: yes/no); and at age of 18 years (BMI z-score, FM and FFM) all of the aforementioned potential confounders were included in addition to: leisure-time and commuting physical activity (minutes/week); smoking status (smoked at least one cigarette in the last 30 days: yes/no); calcium intake (mg/day adjusted for total calories) collected using a semiquantitative food frequency questionnaire; and height (m) and height squared (m^2^) at age 18. We adjusted for height and height squared because the assessment of the model’s quality criteria showed better fit with the inclusion of these two variables.

The present study was approved by the Research Ethics Committee of Universidade Federal de Pelotas School of Medicine. All participants signed a free informed consent form prior to data collection.

## Results

At 18 years 4,106 adolescents (81.3% of the original cohort) were evaluated, of which 2,015 (49.1%) were males. Whole-body BMC and BMD were available for 3,855 participants. Of these, 1,601 males and 1,706 females had complete data on exposure, mediators and confounders, and were therefore included in the analyses. Table 1 shows a comparison between participants included and those not included in the analyses (1,230 losses of follow-up / excluded + 164 deaths + 548 with missing data). Non-white participants and smokers had slightly lower follow up rates than their counterparts did. We also observed lower mean BMI z-score, FM and FFM at age 18 among the male participants as compared to those lost to follow up. Higher family income and a lower proportion of low maternal education (≤8 years) were also observed among females included in the analysis as compared to those lost to follow up.

**Table 1:** **Characteristics of participants with complete data compared with participants with missing data or losses of follow-up, stratified by gender**

**Variables**	**Males**			**Females**		
	**N Mean (SD); percentage**			**N Mean (SD); percentage**		
	**Participants included in the analysis**	**Participants excluded from the analysis** ^**a**^	***p*** **-value**	**Participants included in the analysis**	**Participants excluded from the analysis** ^**a**^	***p*** **-value**
**Follow-up at 11 years**						
Family income	N = 1601	N = 590		N = 1706	N = 555	
	1219.77 (2966.82)	1054.02 (2403.48)	0.224^b^	1062.79 (1597.87)	849.28 (1565.43)	0.006^b^
Maternal education (years)	N = 1601	N = 572		N = 1706	N = 535	
0-4	24.9%	29.6%	0.099^c^	22.6%	35.9%	<0.001^c^
5-8	42.5%	42.1%		43.5%	44.1%	
9-11	22.4%	20.1%		23.5%	13.6%	
≥12	10.2%	8.2%		10.4%	6.4%	
Skin color	N = 1601	N = 509		N = 1706	N = 507	
White	65.5%	60.5%	0.040^c^	65.2%	59.2%	0.013^c^
Non-white	34.5%	39.5%		34.8%	40.8%	
Total physical activity (minutes/week)	N = 1601	N = 515		N = 1706	N = 465	
	514.80 (523.77)	559.49 (563.32)	0.098^b^	347.41 (405.32)	325.42 (367.51)	0.29^b^
Smoking	N = 1601	N = 484		N = 1706	N = 487	
No	97.6%	96.3%	0.129^c^	98.1%	96.7%	0.061^c^
Yes	2.4%	3.7%		1.9%	3.3%	
BMI z score^d^	N = 1601	N = 583		N = 1706	N = 551	
	0.46 (1.22)	0.52 (1.35)	0.349^b^	0.27 (1.23)	0.31 (1.20)	0.531^b^
**Follow-up at 18 years**						
Total physical activity (minutes/week)	N = 1601	N = 407		N = 1706	N = 381	
	855.53 (914.26)	910.45 (913.21)	0.279^b^	455.94 (583.28)	460.20 (521.76)	0.895^b^
Smoking	N = 1601	N = 414		N = 1706	N = 385	
No	86.2%	79.5%	0.001^c^	88.5%	81.0%	<0.001^c^
Yes	13.8%	20.5%		11.5%	19.0%	
BMI z score^d^	N = 1601	N = 372		N = 1706	N = 295	
	0.25 (1.15)	0.45 (1.39)	0.005^b^	0.44 (1.18)	0.47 (1.30)	0.753^b^
Fat mass (kg)	N = 1601	N = 369		N = 1706	N = 297	
	12.46 (8.72)	14.40 (12.60)	<0.001^b^	20.75 (8.90)	21.19 (11.60)	0.453^b^
Fat free mass (kg)	N = 1601	N = 369		N = 1706	N = 297	
	57.74 (7.04)	58.67 (8.25)	0.026^b^	40.15 (4.99)	40.67 (5.41)	0.101^b^
Height (m)	N = 1601	N = 369		N = 1706	N = 298	
	1.74 (0.07)	1.74 (0.08)	0.857^b^	1.61 (0.06)	1.61 (0.06)	0.874^b^
Calcium intake (mg/day)	N = 1601	N = 336		N = 1706	N = 316	
	709.16 (332.71)	726.61 (340.34)	0.384^b^	694.27 (351.62)	679.39 (364.83)	0.492^b^
Bone mineral content (g)	N = 1601	N = 302		N = 1706	N = 246	
	2963.12 (465.24)	2927.32 (441.61)	0.216^b^	2410.69 (396.08)	2411.90 (386.86)	0.964^b^
Bone mineral density (g/cm^2^)	N = 1601	N = 302		N = 1706	N = 246	
	1.22 (0.10)	1.22 (0.09)	0.544^b^	1.13 (0.08)	1.13 (0.08)	0.902^b^

The 1993 Pelotas Birth Cohort. Brazil.

N - Number of observations; SD - Standard deviation; BMI - body mass index

^a^Participants excluded from analysis due to losses of follow-up or missing data.

^b^t-test

^c^Chi-square test

^d^According to the World Health Organization for children and teenagers from 5 to 19 years.

Higher BMI at age 11 was associated with higher BMI, FM and FFM at age 18 in both sexes (see Additional files 1, 2, 3, 4, 5, 6, 7, 8, 9, 10, 11 and 12). At age 18, BMI and FFM were positively associated with BMC and BMD in males and females. FM was positively associated with BMD in females (see Additional file 11) and with BMC in both sexes, and it was inversely associated with BMD in males (see Additional file 5). For example, an increase of 1 kg in FM at age 18 was associated with a BMD 0.002 g/cm^2^ lower among males (95% CI −0.002; −0.001, *p* < 0.001).

Tables 2 and 3 show the overall association between BMI at age 11 and bone mass at age 18 in males and females, respectively, and also, the association after accounting by mediation. BMI at age 11 was associated with greater BMC at age 18 in males by 179.7 g (95% CI 161.4; 198.0, *p* < 0.001) and in females by 179.9 g (95% CI 165.3; 194.6, *p* < 0.001). A positive association was also observed between BMI at age 11 and BMD at age 18 in both sexes (males [β = 0.030 g/cm2, 95% CI 0.024; 0.035, *p* < 0.001]; females [β = 0.029 g/cm2, 95% CI 0.025; 0.033, *p* < 0.001]).

**Table 2:** **Association between body mass index (BMI) z score at 11 years old and bone mass at 18 years mediated by BMI z score, fat mass and fat free mass at 18 years among men**

**Association entre BMI z score at 11 y and BMC/BMD**	**BMC (g)**		**BMD (g/cm** ^**2**^ **)**	
	**β (95% CI)p-value**		**β (95% CI)p-value**	
	**Association**	**% mediation**	**Association**	**% mediation**
Overall association^(A)^	179.714(161.401; 198.027)*p* < 0.001	n/a	0.030(0.024; 0.035)*p* < 0.001	n/a
Association after accounting for mediation by BMI z score at 18 years old^(B)^	25.764(8.536; 42.993)*p* = 0.003	85.7	0.005(−0.0004; 0.010)*p* = 0.068	83.3
Association after accounting for mediation by fat mass at 18 years old^(B)^	167.344(151.041; 183.648)*p* < 0.001	6.9	0.037(0.032; 0.041)*p* < 0.001	-^a^
Association after accounting for mediation by fat free mass at 18 years old^(B)^	38.820(27.874; 49.766)*p* < 0.001	78.4	−0.003(−0.007; −0.0001)*p* = 0.044	90.0
Association after accounting for mediation by BMI z score, fat mass and fat free mass at 18 years old^(B)^	20. 375(6.717; 34.033)*p* = 0.003	88.7	0.003(−0.001; 0.007)*p =* 0.160	90.0

The 1993 Pelotas Birth Cohort. Brazil. N = 1601 males.

β - Linear regression coefficient; 95% CI - 95% confidence interval; p-value from Wald’s test; BMC - bone mineral content; BMD - bone mineral density; BMI - body mass index; BMI z score: according to the World Health Organization for children and teenagers from 5 to 19 years.

% mediation = 100 - (Direct effect/Total effect)*100, ie, 100 - (B/A)*100

^a^Percentage of mediation cannot be calculated because of the negative association of FM with BMD in males.

**Table 3:** **Association between body mass index (BMI) z score at 11 years old and bone mass at 18 years mediated by BMI z score, fat mass and fat free mass at 18 years among females**

**Association entre BMI z score at 11 y and BMC/BMD**	**BMC (g)**		**BMD (g/cm** ^**2**^ **)**	
	**β (95% CI)p-value**		**β (95% CI)p-value**	
	**Association**	**% mediation**	**Association**	**% mediation**
Overall association^(A)^	179.945(165.306; 194.584)*p* < 0.001	n/a	0.029(0.025; 0.033)*p* < 0.001	n/a
Association after accounting for mediation by BMI z score at 18 years old^(B)^	56.571(42.714; 70.428)*p* < 0.001	68.6	0.011(0.007; 0.015)*p* < 0.001	62.1
Association after accounting for mediation by fat mass at 18 years old^(B)^	122.570(109.272; 135.869)*p* < 0.001	31.9	0.025(0.022; 0.029)*p* < 0.001	13.8
Association after accounting for mediation by fat free mass at 18 years old^(B)^	73.100(61.975; 84.225)*p* < 0.001	59.4	0.003(0.0004; 0.006)*p* = 0.024	89.7
Association after accounting for mediation by BMI z score, fat mass and fat free mass at 18 years old^(B)^	43.008(30.140; 55.875)*p* < 0.001	76.1	0.007(0.003; 0.010)*p* < 0.001	75.9

The 1993 Pelotas Birth Cohort. Brazil. N = 1706 females.

β - Linear regression coefficient; 95% CI - 95% confidence interval; p-value from Wald’s test; BMC - bone mineral content; BMD - bone mineral density; BMI - body mass index; BMI z score: according to the World Health Organization for children and teenagers from 5 to 19 years.

% mediation = 100 - (Direct effect/Total effect)*100, ie, 100 - (B/A)*100

BMI and FFM at age 18 were found to be strong mediators of the association between BMI at age 11 and bone mass; in almost all cases, the positive association between BMI at age 11 and BMC/BMD was reduced after taking these pathways into account (Tables 2 and 3). In males, both BMI and FFM at age 18 years were key mediators. For example, after the inclusion of these two variables in the model, 85.7% and 78.4%, respectively, of the total effect of BMI at age 11 on BMC, was mediated by the model (Table 2). Among men, 83.3% of the association between BMI at age 11 and BMD was mediated by BMI and 90.0% by FFM. In females (Table 3), the mediation was less marked; BMI at age 18 acted as the key mediator in the association between BMI at age 11 and BMC (68.6% of the total effect) and FFM was the key mediator in the association with BMD (89.7%).

FM was not found to be a strong mediator of the association between BMI at age 11 and BMC/BMD at age 18. In females, the percentage mediation was much lower than for BMI or FFM; 31.9% for BMC and 13.8% for BMD. In males, the percent mediation was 6.9% for the association between BMI at age 11 and BMC at age 18. FM had a negative association with BMD in males (see Additional file 5), and as such did not mediate the association between BMI at age 11 and BMD at age 18.

## Discussion

Our findings point to a positive association between BMI at age 11 and both BMC and BMD at age 18, with this association being largely mediated by contemporaneous body composition at age 18, in particular by BMI and FFM. Recent scientific evidence has shown that greater body weight or BMI correlate positively with BMD and BMC [2,5]. In this study, we have demonstrated a positive association between BMI at two different time points in adolescence and BMC/BMD at age 18. One potential explanation for this finding is that higher BMI may lead to an increased osteogenesis resulting from the mechanical load exerted by excessive weight on bone structure [2].

We found a positive association of FFM with BMC/BMD in both sexes, which corroborates the findings of other studies [20-22] and of a recent meta-analysis [23]. Several hypotheses have been postulated to explain the positive association between FFM and bone mass. The most robust hypothesis is that muscles and other fat-free soft tissues exert mechanical loads on bone tissue [24]. Additional muscle and FFM due to either greater physical activity or greater total body mass can promote increased bone mass [25-27].

We also found a negative association of FM with BMD in males, corroborating the findings of a recent meta-analysis [23] and of other studies with adults and adolescents [10,28,29]. However, it is noteworthy that a positive association with BMC/BMD was observed in females in our analyses. Some studies have reported higher FM associated with increased bone mass among females [10]. Several mechanisms could explain the relationship between FM and bone mass: e.g. mechanical load on bone tissue exerted by fatty soft tissues, or the association of FM with hormones secreted from pancreatic beta cells (insulin and amylin) and adipocytes (estrogens and leptin) that have a direct or indirect effect on osteogenesis [24,30-32]. It should also be stressed that during adolescence girls gain more FM while boys gain more lean mass [33]. The difference in body composition of males and females may possibly explain this positive association of FM with BMC observed only among females.

All variables studied here (BMI, FM and FFM at age 18) acted to some extent as mediators of the association of BMI in early adolescence with BMC and BMD at age 18. There were major differences between males and females. Among men, it was observed that the association between BMI at 11 years and BMD was completely mediated by BMI, FM and FFM at 18 years; the inclusion of these mediators strongly attenuated the association towards the null hypothesis; the same was not observed for BMC. This suggests that, at least in males, BMI in early adolescence is not a major determinant of bone mineral density later in life, other than through ‘tracking’, i.e. individuals with high BMI at age 11 tend to remain at high BMI across the life course.

In females, BMI, FM and FFM at age 18 partially explained the association between BMI in early adolescence and BMC/BMD, but there remained an association between BMI at age 11 and BMC/BMD even after controlling for the mediators. Therefore, in contrast to the findings in males, BMI in early adolescence may be a determinant of bone mass at age 18 in females independent of concurrent body size and composition. These differences of males and females may be attributed to the effect of hormones on body composition and bone mass acquisition. However, the 1993 Pelotas birth cohort did not provide accurate sexual maturation data including sex hormone levels, and so this hypothesis cannot be further evaluated within this population. Boys and girls do not show the same timing of changes in body composition and bone development resulting from sexual maturation [33]. Girls typically start puberty two years earlier than boys [33], and by age 11 have entered puberty while boys still are prepubertal [33]. A possible explanation to our finding is that BMI at age 11 is a marker for pubertal status in females but not males, and that age of puberty onset influences BMC and BMD.

To the best of our knowledge, this is the first study conducted with data from a prospective birth cohort that sought to examine the association between BMI at different time points in adolescence, FM, FFM and bone mass in late adolescence, using path analysis. Path analysis is an extension of multiple regression that facilitates modeling the relationships among all variables simultaneously, facilitating the analysis of pathways and mediation [34]. Another strength of the present study was that BMC and BMD measures were obtained by DXA, which is the gold-standard method for measuring bone mass [35]. Despite its innovative approach, the study has a number of limitations. First, as with all longitudinal studies, we had follow up losses of participants. However, we believe these losses did not affect our results, because there were no significant differences for most variables between participants included and those not included in the analysis, and we have no reason to expect that the association between BMI at age 11 and BMC/BMD at age 18 would differ between those included and those excluded from our analyses. Second, there was no information about vitamin D status, which plays a major role in the intestinal absorption of calcium, and as such we are unable to explore this. The lack of information on vitamin D and on sexual maturation are the most important limitations of the present paper for understanding the association between BMI at the beginning of adolescence and BMC/BMD at 18 years old. The next follow-ups of this cohort will add important contributions on this issue.

## Conclusions

This study found a positive association of BMI at age 11 with BMC and BMD at age 18. This association was partially mediated by BMI and FFM at age 18, particularly in males. FM was not an important mediator of the association between BMI at 11 and BMC/BMD at 18 years. Our findings suggest that concurrent BMI and FFM are the main mediators of the association between BMC and BMD in late adolescence and BMI in earlier adolescence.

## Additional files

Additional file 1: Figure S1. Overall association between BMI z score at 11 and 18 years and bone mineral content at age18 among males (N = 1601). The 1993 Pelotas Birth Cohort. Brazil. Additional file 2: Figure S2. Overall association between BMI z score at 11, fat mass and bone mineral content at age18 among males (N = 1601). The 1993 Pelotas Birth Cohort. Brazil. Additional file 3: Figure S3. Overall association between BMI z score at 11, fat free mass and bone mineral content at age18 among males (N = 1601). The 1993 Pelotas Birth Cohort. Brazil. Additional file 4: Figure S4. Overall association between BMI z score at 11 and 18 years and bone mineral density at age18 among males (N = 1601). The 1993 Pelotas Birth Cohort. Brazil. Additional file 5: Figure S5. Overall association between BMI z score at 11, fat mass and bone mineral density at age18 among males (N = 1601). The 1993 Pelotas Birth Cohort. Brazil. Additional file 6: Figure S6. Overall association between BMI z score at 11, fat free mass and bone mineral density at age18 among males (N = 1601). The 1993 Pelotas Birth Cohort. Brazil. Additional file 7: Figure S7. Overall association between BMI z score at 11 and 18 years and bone mineral content at age18 among females (N = 1706). The 1993 Pelotas Birth Cohort. Brazil. Additional file 8: Figure S8. Overall association between BMI z score at 11, fat mass and bone mineral content at age18 among females (N = 1706). The 1993 Pelotas Birth Cohort. Brazil. Additional file 9: Figure S9. Overall association between BMI z score at 11, fat free mass and bone mineral content at age18 among females (N = 1706). The 1993 Pelotas Birth Cohort. Brazil. Additional file 10: Figure S10 Overall association between BMI z score at 11 and 18 years and bone mineral density at age18 among females (N = 1706). The 1993 Pelotas Birth Cohort. Brazil. Additional file 11: Figure S11. Overall association between BMI z score at 11, fat mass and bone mineral density at age18 among females (N = 1706). The 1993 Pelotas Birth Cohort. Brazil. Additional file 12: Figure S12. Overall association between BMI z score at 11, fat free mass and bone mineral density at age18 among females (N = 1706). The 1993 Pelotas Birth Cohort Brazil.

## Abbreviations

BMI: Body mass index
FM: Fat mass
FFM: Fat free mass
BMC: Bone mineral content
BMD: Bone mineral density
DXA: Dual-energy x-ray absorptiometry
