# Supplementary material for: Body mass index at 11 years and bone mass at age 18: path analysis within the 1993 Pelotas (Brazil) birth cohort study
Source: BMC Musculoskelet Disord. 2015 Mar 29;16:71. doi: 10.1186/s12891-015-0529-y (PMC4391135; doi:10.1186/s12891-015-0529-y)
Supplement: Additional file 7: Figure S7. — Overall association between BMI z score at 11 and 18 years and bone mineral content at age18 among females (N = 1706). The 1993 Pelotas Birth Cohort. Brazil. [file 12891_2015_529_MOESM7_ESM.pdf]

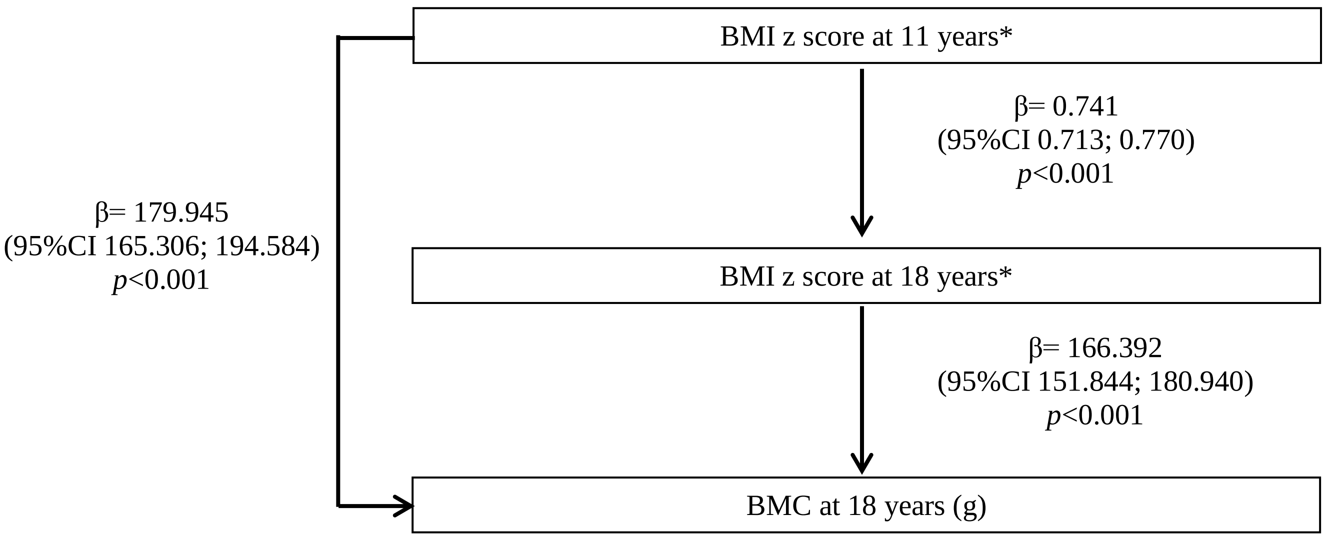

Figure 7 Overall association between BMI z score at 11 and 18 years and bone mineral content at age18 among females (N=1706). The 1993 Pelotas Birth Cohort. Brazil.

β - linear regression coefficient; 95%CI - 95% confidence interval; *p*-value from Wald's test; BMI - body mass index; BMC - bone mineral content; *According to the World Health Organization for children and teenagers from 5 to 19 years
